# Alkalistable endo-β-1,4-xylanase production from a newly isolated alkalitolerant *Penicillium* sp. SS1 using agro-residues

**DOI:** 10.1007/s13205-011-0009-5

**Published:** 2011-06-07

**Authors:** Bijender Kumar Bajaj, Mukul Sharma, Sunny Sharma

**Affiliations:** 1 School of Biotechnology, University of Jammu, Jammu, 180 006 India; 2 Present Address: Biotechnology and Fermentation Group, Department of Animal Sciences, Gerlaugh Hall, Ohio Agricultural Research and Development Centre (OARDC), The Ohio State University, 1680 Madison Avenue, Wooster, OH 44691 USA

**Keywords:** Xylanase, *Penicillium* sp., Alkalistable, Thermoactive, Wheat bran, Rice bran

## Abstract

Thermostable and alkalitolerant xylanases have got intense research focus due to their vast applications in various industries including pulp and paper, food, feed, textile, biofuel, etc. In the present investigation, a *Penicillum* sp. SS1 isolated from degrading woody material was found to produce moderately thermoactive and alkalistable endo-β-1,4-xylanase (xylanase). Maximum xylanase production was observed after fourth day of fermentation (43.84 IU/ml). The organism produced substantial quantities of xylanase using agricultural residues like wheat bran (20.6 IU/ml), rice bran (21.8 IU/ml) and sawdust (10.7 IU/ml) as carbon sources. The enzyme preparation was totally free of filter paper activity (FPase) and possessed negligible carboxymethyl cellulase (CMCase) activity; this could be an important feature of enzyme if the intended application of enzyme is in pulp and paper industries. Among nitrogen sources examined, yeast extract supported maximum xylanase production (45.74 IU/ml), and was followed by soybean meal (22.2 IU/ml) and ammonium sulphate (20 IU/ml). Maximum xylanase production was observed at initial medium pH 9 (25.6 IU/ml); however, at pH 8 and 10 also significantly high enzyme titre was observed (24 and 21.2 IU/ml, respectively). Thus, *Penicillium* sp. SS1 displayed capability of growing and producing xylanase at high alkaline pH (8–10). Maximum xylanase activity was reported at 50 °C, however, significantly high activity was observed at 60 °C (65.4%), however, at 70–80 °C activity was lost considerably. At 50–60 °C the enzyme retained very high activity up to 30–60 min (91–100%), however, prolonged incubation (90 min) caused considerable activity reduction (residual activity 63–68%).

## Introduction

In recent years, increasing concern over preserving natural resources and the environment has initiated a growing interest in application of microbially produced enzymes in industrial processes. Xylan degrading enzymes have attracted much attention because of their applications in industrial processes such as the modification of cereal-based food stuffs, improving the digestibility of animal feed stocks, bioconversion of lignocellulosic materials and agro-wastes to fermentable products, and pre-bleaching of paper pulps (Kuhad and Singh 1993; Beg et al. 2001). Xylanases are glycosidases (*O*-glycoside hydrolases, EC 3.2.1.X) which catalyze the endohydrolysis of 1,4-β-D xylosidic linkages in xylan. The complete hydrolysis of xylan involves the synergistic action of an array of main and side-chain-cleaving enzymes including endo-β-1,4-xylanase, β-d-xylosidase, α-1-arabinofuranosidase, α-glucuronidase and acetyl xylan esterase, but hydrolysis by endo-β-1,4-xylanase (or simply xylanase) is considered as one of the key reactions in the process (Kuhad and Singh 1993; Beg et al. 2001). As hemicelluloses are the second most abundant renewable resource, and xylan constitutes the major component of hemicelluloses, therefore, xylanolytic enzymes derived from microbial sources could be of great industrial, environmental and strategic significance (Sharma and Bajaj 2005; Sudan and Bajaj 2007; Liu et al. 2008; Bajaj and Singh 2010; Nagar et al. 2011). The increasing interest in xylanases in recent years has mainly been due to their potential applications in the pulp and fiber processing industries. Xylanases promote bleaching through the hydrolysis of relocated and re-precipitated xylan on the surface of the pulp fibre, this results in better chemical penetration and consequently improved lignin extractability. The application of xylanases in pulp and paper reduces the need for toxic chemicals such as chlorine and chlorine dioxide, during the bleaching process of pulps and, thus, promote the development of environment-friendly industrial process (Singh et al. 2011). Prebleaching of the paper pulp requires the use of cellulase-free xylanases, since cellulases may adversely affect the quality of the paper pulp by destroying the structure of cellulose and, thus, diminish the quality of pulp (Kuhad and Singh 1993; Beg et al. 2001).

For successful industrial applications, xylanases must be thermostable, alkalistable and robust so that they can withstand the harsh industrial process conditions. Most of the xylanases characterized so for show optimal activity at acidic, neutral or slightly alkaline pH (7–8) and at temperature between 40 and 60 °C. In the search for thermostable and alkalistable xylanases, microbial diversity has been extensively exploited (Bajaj and Singh 2010; Singh et al. 2011; Nagar et al. 2011). Furthermore, utilization of low-cost agriculture-based crude substrates can play a crucial role in overall cost-reduction for bulk production of xylanases for industrial applications.

Keeping in view the importance of thermostable and alkalistable xylanases in various industrial sectors, the present investigation was aimed at production and characterization of thermoactive and alkalistable xylanase from a newly isolated alkalitolerant *Penicillium* sp. SS1.

## Materials and methods

### Isolation of xylanolytic fungi

For isolation of fungi, samples (alkaline soil, cow-dung manure, degrading sawdust, bagasse, poultry waste etc.) were suspended (5%, w/v) in normal saline, and suspension was appropriately diluted and spread plated (100 μl) on PDA (potato-dextrose-agar) and plates were incubated at 30 °C for 4 days. The fungal colonies appeared were examined microscopically and purified by re-streaking on PDA. All the fungal isolates were maintained on PDA slants at 4 °C.

For analyzing xylanolytic activity of the isolated fungi, all the isolates were spotted (2–3 mm) on the xylan agar (each, %, w/v, ammonium sulphate 0.3, potassium dihydrogen phosphate 0.3, ammonium acetate 0.6, oat spelt xylan 0.5, agar 2; pH 8–10). The plates were incubated at 30 °C for 4 days. Colonies developed were assayed for xylanase producing ability by Congo red staining (Sudan and Bajaj 2007). The colonies were flooded with 0.1% (w/v) Congo red for 15 min and then washed three times with 1 M NaCl for 15 min each. Fungal colonies showing clear zones around them were considered positive for xylanase production, and the one (isolate SS1, identified as *Penicillium* sp. and designated as *Penicillium* sp. SS1) which showed the biggest halo was selected and further subjected to submerged fermentation for xylanase production.

### Fermentation for analyzing growth and enzyme production

The selected organism *Penicillium* sp. SS1 was grown on PDA plates for 4 days and three discs (2 mm, each) of fungal biomass were cut and inoculated into 100 ml of xylanase production medium (same composition as of above mentioned xylan agar but minus agar, and pH 7–10) contained in Erlenmeyer flasks of 250 ml capacity. Flasks were incubated on a shaker-cum incubator (Innova, New Brunswick, USA) at 30 °C (180 rpm). After suitable intervals of time, samples were withdrawn, centrifuged at 10,000×*g* for 5 min (Sigma 3K30, UK) and supernatant was considered to be equivalent to crude enzyme and was used for assaying xylanase activity. For determining the growth of the organism, the mycelial mass was filtered through Whatman no. 1 filter paper, and the biomass obtained was weighed after appropriately drying it.

### Determination of inducible/constitutive nature of xylanase, and exo/endoxylanolytic activity

Fermentation was conducted in xylanase production medium, and in the medium in which xylan of the production medium was replaced with glucose (0.5%, w/v), at 30 °C under shaking (180 rpm) for 96 h. The fermentation broth was centrifuged and the supernatant from both the media was used for assaying the xylanase activity. The supernatant was loaded at the rate of 50 μl in the wells (diameter, 7 mm) cut in the xylan agar plates. The plates were incubated for 2–4 h, and then subjected to Congo red staining.

The exo/endoxylanolytic activity of the *Penicillium* sp. SS1 xylanase was examined by carrying out thin layer chromatography (TLC) on silica gel plates (Kosugi et al. 2001). The spots of standard xylose, standard xylan, and xylanase-treated xylan (1 ml of 0.5% xylan was incubated with 200 μl of xylanase for 24 h at 37 °C) were applied, and TLC was run using acetone–ethyl acetate–acetic acid (2:1:1, v/v/v) as solvent system. The plates were visualized by spraying with 1:1 (v/v) mixture of 0.2% methanolic orcinol and 20% sulphuric acid.

### Xylanase, carboxymethyl cellulase (CMCase) and FPase assay

Xylanase activity was assayed using 0.5% xylan in suitable buffer (Tris buffer, 50 mM, pH 8) as the substrate at 45 °C. The reducing sugars released were assayed by dinitrosalicylic acid (DNSA) method using xylose as standard (Miller 1959). One unit of enzyme activity (International Unit, IU) is defined as the enzyme necessary to release 1 μmol of reducing sugar or xylose equivalent per min under assay conditions.

In the same enzyme preparation CMCase and FPase were also assayed by employing DNSA method using carboxymethyl cellulose and filter paper as substrates, respectively (Sharma and Bajaj 2005; Sudan and Bajaj 2007). One unit (IU) of CMCase or FPase was defined as the amount of enzyme required to release 1 μmol of reducing sugar equivalent per min under the assay conditions.

### Xylanase production using alternate carbon and nitrogen sources

To study the effect of agriculture-based crude carbon sources on xylanase production the xylan of the xylanase production medium was replaced with alternatives like wheat bran, rice bran or sawdust (1%, w/v), as sole carbon source in the medium. Crude substrates were first crushed and then steam hydrolyzed by autoclaving (15 psi for 15 min) and then used in the medium (1%, w/v). Similarly, to analyze the effect of alternative nitrogen sources on xylanase production, the normal nitrogen source of the production medium was replaced with alternative nitrogen sources viz. yeast extract, urea, soybean meal or peptone (0.5% w/v), and fermentation was conducted as usual.

### Effect of initial medium pH on xylanase production

The initial pH of xylanase production medium containing xylan (1%, w/v) as carbon source and yeast extract (0.5%, w/v) as nitrogen source, was adjusted to 8, 9 or 10 using Na_2_CO_3_ (2%, w/v) and fermentation was conducted.

### Effect of temperature and pH on xylanase activity, and thermostability of xylanase

For studying the effect of temperature on enzyme activity, the enzyme assay mixture was incubated at different temperatures (30–90 °C) and enzyme activity was determined. For determining effect of pH on enzyme activity, different buffers (50 mM) were used in the assay mixture (citrate buffer for pH 3, 4, 5 and 6; tris buffer for pH 7, 8 and 9; glycine-NaOH buffer for pH 10) and activity was assayed. The thermostability of the enzyme was tested by pre-incubating the enzyme preparation at various temperatures (50–90 °C) for different intervals of time (30–90 min) and then assaying the residual activity.

All the analytical experiments were set in triplicates and the results represent the mean of three different experiments, standard deviation was determined and coefficient of variation was within 10%.

## Results and discussion

### Xylanolytic organism

Of the 23 fungal isolates examined, the isolate SS1 produced the largest halo on xylan agar plates upon Congo red staining. Therefore, this isolate was selected for further studies. The isolate SS1 formed powdery colonies on xylan agar plate. Colonies were yellow coloured after 4 days of incubation and reverse was dark yellow. Microscopic examination of the morphology of the organism revealed that it was *Penicillium* sp., and the organism was designated as *Penicillium* sp. SS1.

### Time course of growth and xylanase production

**Fig. 1:**
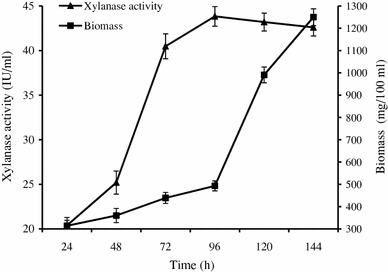
Growth and time-course of xylanase production by *Penicillium* sp. SS1. Fermentation was conducted in production medium under shaking (180 rpm) at 30 °C, and biomass and xylanase produced were assayed. *Error bars* show standard deviations

Xylanase production and growth were studied at 30 °C for 6 days in production medium (pH 8) containing 1% xylan as carbon source. It was found that xylanase production started from second day and reached a maximum on the fourth day (43.84 IU/ml), and then remained almost constant (Fig. 1). The growth and xylanase production went parallel up to fourth day but after that no further increase in enzyme production was observed, however, growth kept on increasing till sixth day. Similar trend was observed when growth and xylanase production were examined at pH 7.0, i.e. maximum enzyme production occurred at fourth day of fermentation (43.81 IU/ml) while increase in growth continued up to sixth day (data not shown). Most of the earlier reports indicate that maximum xylanase production by fungi occur after 4–5 days of fermentation (Carmona et al. 1998; Ruckmanl and Rajendran 2001; Shah and Madamwar 2005; Sudan and Bajaj 2007; Murthy and Naidu 2010). However, *Penicillium* sp. ZH-30 produced maximum xylanase after 3 days of fermentation (Li et al. 2007). It would appear that the maximum enzyme production stage of any organism is largely dependent upon the type of microbial strain and its genetic make-up as well as on cultural and environmental conditions during the growth of the organism.

### Inducible and endoxylanolytic nature of xylanase

**Fig. 2:**
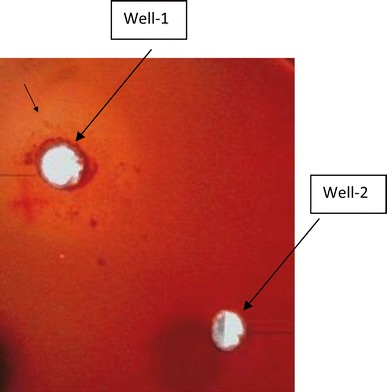
Demonstration of inducible nature of *Penicillum* sp. SS1 xylanase on xylan agar plate by Congo red staining. Well-1 was loaded with cultural supernatant from medium with xylan as carbon source, well-2 was loaded with cultural supernatant from medium with glucose as carbon source. *Arrow* indicates zone of clearance

**Fig. 3:**
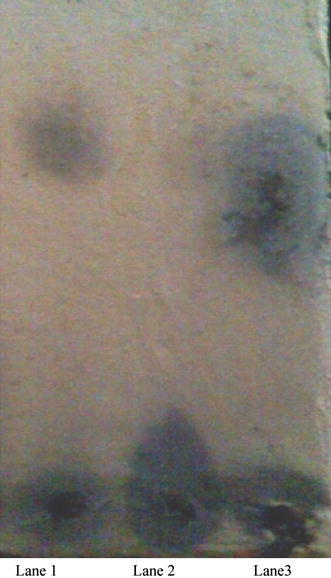
Demonstration of endoxylanolytic nature of *Penicillium*sp. SS1 xylanase.*Lane 1* and*lane 2* indicate standard xylose control and standard xylan control, respectively, while the*lane 3* represents xylanase-treated xylan, showing the presence of xylan degradation products (xylose, xylobiose, xylotriose and xylooligosaccharides) due to action of xylanase on xylan

When the xylan of the production medium was replaced with glucose, no xylanase production was observed as indicated by the absence of clear zone upon Congo red staining on xylan agar, however, as expected enzyme was produced when organism was cultivated in medium with xylan as carbon source (Fig. 2). Thus, the *Penicillium* sp. SS1 xylanase is inducible in nature and not of constitutive nature. Most of the microbial xylanases reported are of inducible in nature (Shah and Madamwar 2005; Sudan and Bajaj 2007; Singh et al. 2011), however, constitutive xylanases are also known (Bocchini et al. 2008). Furthermore, the *Penicillium* sp. SS1 xylanase was found to be an endoxylanase (Fig. 3). The spots of standard xylose, standard xylan, and xylanase-treated xylan were applied on TLC plate. Xylan did not move much because of its high molecular weight, xylose being of the lowest molecular weight moved ahead among all, while xylanase-treated xylan showed movement which was in between the xylan and xylose. These results indicate that the xylan has been cleaved endolytically by xylanase and may have produced a mixture of xylose, xylobiose, xylotriose and xylooligomers. Similarly, *Clostridium cellulovorans* xylanase has been reported to produce a mixture of xylose, xylobiose, xylotriose and xylooligomers from xylan (Kosugi et al. 2001).

### Xylanase production using agriculture-based crude carbon sources

**Fig. 4:**
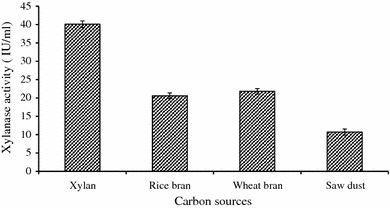
Xylanase production by *Penicillium* sp. SS1 on agriculture-based carbon sources. Xylan of the production medium was replaced with either of the carbon source and fermentation was conducted (180 rpm) at 30 °C. *Error bars* indicate standard deviations

Since the cost of the substrate plays a crucial role in the economics of any industrial production process, therefore, low-value crude agriculture-based raw materials may be employed as cost-effective substrates for xylanase production. India being blessed with rich agricultural heritage there is no dearth of agricultural-based residues. Utilization of agricultural residues for enzyme production may serve dual purpose, on one side it provides a valuable product (enzyme) and on the other side also help solving environmental waste disposal problem. In the present study, various carbon sources viz. wheat bran, rice bran and sawdust were employed as substrates for xylanase production. *Penicillium* sp. SS1, although showed the maximum xylanase production on xylan (40.1 IU/ml), but substantial xylanase activity was observed on crude carbon sources like wheat bran (21.8 IU/ml), rice bran (20.6 IU/ml) and sawdust (10.7 IU/ml), as well (Fig. 4). Maximum xylanase production was reported on the fourth day of cultivation in all the cases.

When the xylanases are to be used for the pulp and paper industries then the enzyme preparations must be free of cellulases else the cellulose fibres may be damaged. In the present study, the enzyme preparation did not show FPase activity at all, however, displayed negligible carboxymethyl cellulase activity (0.088 IU/ml). Thus, such enzyme preparation may have potential to be used in pulp and paper industries. Many researchers have reported moderate or negligible or even a total absence of cellulase activity in xylanase preparation (Bakir et al. 2001). However, xylanases with cellulase activity can be successfully used for alternative applications, i.e. for the food and feed industry, lignocellulose biotransformation industries and in silage preparation (Sharma and Bajaj 2005). There are many reports on xylanase production using agricultural based crude carbon sources (Bakri et al. 2003; Min et al. 2007; Sudan and Bajaj 2007; Bajaj and Singh 2010; Singh et al. 2011). Rice husk and wheat bran were found to be good substrates for xylanase production by *Aspergillus niveus* RS2 (Sudan and Bajaj 2007). Rice straw and wheat bran in ratio of 3:1 proved to be the best substrate for maximum xylanase production from *A. niger* KK2 (Min et al. 2007). *Streptomyces* sp. 7b showed maximum xylanase production on wheat bran (Bajaj and Singh 2010).

### Effect of nitrogen sources on xylanase production

**Fig. 5:**
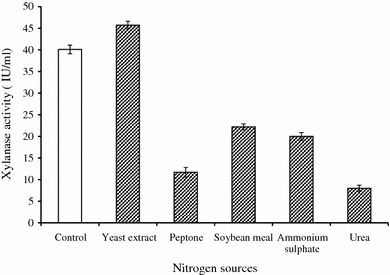
Xylanase production by *Penicillium* sp. SS1 using different nitrogen sources. The usual nitrogen source of the production medium was replaced with either of the nitrogen source and fermentation was conducted. *Error bars* show standard deviations

Formation of extracellular enzymes is influenced by the availability of nitrogen source as it is the ultimate precursor for protein synthesis. Furthermore, nitrogen source can significantly affect the pH of medium during the course of fermentation, and hence enzyme activity and stability may also get influenced. In the present study, the usual nitrogen source of the production medium was replaced with different alternative nitrogen sources viz. yeast extract, urea, soybean meal or peptone (0.5%, w/v). Among these, the yeast extract supported maximum enzyme production (45.74 IU/ml) on fourth day of fermentation. Soybean meal and ammonium sulphate also resulted in significantly high enzyme activity (22.2 and 20 IU/ml, respectively), however, urea and peptone as nitrogen sources caused repression in xylanase synthesis (Fig. 5). Similar to above results, yeast extract has been reported as one of the best nitrogen source for xylanase production by different fungal species (Sudan and Bajaj 2007; Min et al. 2007). In contrast, Sharma and Bajaj (2005) reported that soybean meal is the best nitrogen source while yeast extract and gelatin are moderately good for xylanase production by *Streptomyces* sp. CD3. In another study, tryptone supported higher xylanase production as compared to yeast extract and soybean meal (Bajaj and Singh 2010). Combination of yeast extract and peptone were found to be the best for maximum xylanase production by *Penicillium canescens* 10-10c (Bakri et al. 2003). Urea has been reported as recalcitrant source of nitrogen for xylanase production as it leads to repression in xylanase biosynthesis while soy flour has been found to be the most conditioned nitrogen source (Bakri et al. 2003).

### Effect of initial medium pH on xylanase production

**Fig. 6:**
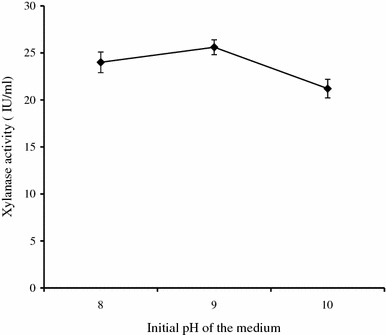
Effect of initial medium pH on xylanase production by *Penicillium* sp. SS1. pH of the production medium was adjusted with sodium carbonate (2%, w/v) and fermentation was conducted at 30 °C on shaker (180 rpm). *Error bars* show standard deviations

For studying the effect of initial pH of medium on xylanase titre, production medium with 1% xylan and 0.5% yeast extract was used and adjusted at pH 8, 9 and 10. Maximum xylanase activity was observed in medium at pH 8 and 9, i.e. 24 and 25.6 IU/ml, respectively, while at pH 10, xylanase activity was slightly decreased, i.e. 21.2 IU/ml (Fig. 6). The results indicate that the selected organism *Penicillium* sp. SS1 is quite alkalitolerant and is not only capable of growing well but also producing sufficiently high xylanase titre at high alkaline pH. Majority of fungal species have been reported to produce maximum xylanase at acidic pH (Carmona et al. 1998; Subramanian and Prema 2000). There are reports of xylanase production at alkaline pH also. *A. niveus* RS2 showed a greater xylanase production at a neutral and slightly alkaline pH (pH 8) than at an acidic and high alkaline pH (Sudan and Bajaj 2007). However, Ruckmanl and Rajendran (2001) reported maximum xylanase production from *A. flavus* at pH 9.

### Effect of pH on enzyme activity

**Fig. 7:**
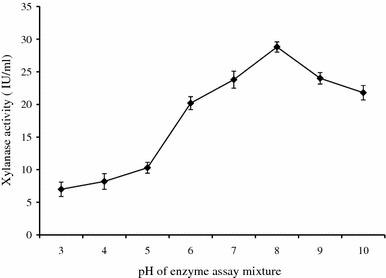
Effect of pH on activity of *Penicillium* sp. SS1 xylanase. Xylanase assay was performed at different pH (3–10) using appropriate buffers. *Error bars* indicate standard deviations

The majority of xylanases reported to date are optimally active in the acidic, neutral or slight alkaline pH range (Subramanian and Prema 2000; Sudan and Bajaj 2007). From the application point of view, xylanases that are active and stable at alkaline pH range are important. Xylanase assay was conducted at different pH by employing appropriate buffers. Maximum xylanase activity was observed at pH 8 (28.8 IU/ml) and it decreased slightly at pH 7 (23.8 IU/ml), however, substantial amount of enzyme activity was retained under high alkalinity, i.e. 24 and 21.8 IU/ml at pH 9 and pH 10, respectively, as shown in Fig. 7. Results indicate that enzyme is highly alkalitolerant and may comply with the industrial processes (pulp and paper) which are carried out at higher pH. Xylanase from *P. citrinum* showed pH optima of 8.5 (Dutta et al. 2007) while that from *Talaromycesthermophilus* was maximally active at pH 7–8 (Maalej et al. 2009).

### Effect of temperature on enzyme activity

**Fig. 8:**
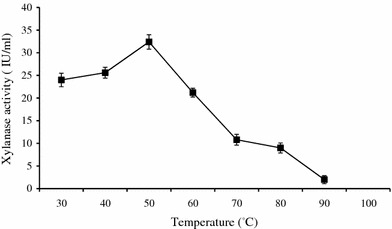
Effect of temperature on activity of *Penicillium* sp. SS1 xylanase. Xylanase assay was conducted at different temperatures (30–90 °C). *Error bars* show standard deviations

For studying the effect of temperature on enzyme activity, the enzyme assay was carried out at different temperatures (30–90 °C). Maximum xylanase activity was reported at 50 °C (32.4 IU/ml), however, substantially high activity was observed at 60 °C (21 IU/ml) as presented in Fig. 8. But at still higher temperatures (70–90 °C) activity decreased. As most of the industrial processes are accomplished at quite higher temperatures, therefore, it is highly desirable that the enzyme preparation intended to be used for such processes must be capable of withstanding high temperatures. Fungal xylanases typically have temperature optima of about 50 °C (Ruckmanl and Rajendran 2001; Shah and Madamwar 2005; Sudan and Bajaj 2007; Dutta et al. 2007; Murthy and Naidu 2010). However, the xylanases with higher temperature optima have also been reported which could have potential to be used in paper and pulp industry. *T.thermophilus* xylanase has been reported to have temperature optima of 75–80 °C (Maalej et al. 2009).

### Thermostability of xylanase

**Fig. 9:**
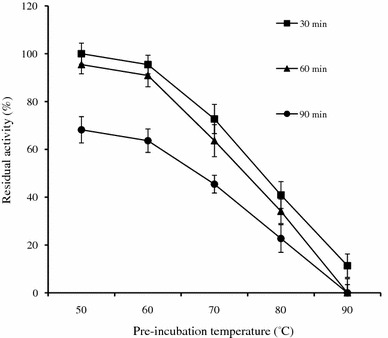
Thermostability of xylanase from *Penicillium* sp. SS1. Enzyme was preincubated at different temperatures (50–90 °C) for varying time periods (30–90 min) and then assayed for residual activity. *Error bars* show standard deviations

The enzyme preparation was pre-incubated at temperatures ranging from 50 to 90 °C for different time periods (30–90 min) and residual activity was assayed. At 50–60 °C the enzyme retained very high activity up to 30–60 min (91–100%), however, prolonged incubation (90 min) caused considerable activity reduction (residual activity 63–68%) as shown in Fig. 9. At 70 °C xylanase showed substantial activity (63–72%) up to 30–60 min but considerable activity reduction occurred after 90 min (residual activity 45%). Pre-incubation of enzyme at still higher temperature (80 °C) resulted in residual activity of 34–40% after 30–60 min, while after 90 min residual activity was 23%. At 90 °C almost complete inactivation of xylanase was observed (Fig. 9). Substantial stability of the xylanase at elevated temperature reflects that enzyme could have potential industrial significance particularly in processes which are accomplished at higher temperatures. *A. foetidus* xylanase retained residual activity of 71 and 20% after 30 min of exposure at 50 and 60 °C, respectively, while at 70 °C the enzyme was completely inactivated within 30 min (Shah and Madamwar 2005). *A. versicolor* xylanase I and xylanase II showed half-lives of 17 and 1.7 min, respectively, at 60 °C (Carmona et al. 1998). Similarly, Sudan and Bajaj (2007) reported that *A. niveus* RS2 xylanase retained 97, 88.9 and 70.9% of the initial activity after 20, 30 and 40 min of incubation at 50 °C, respectively, while at 60 °C the residual activity after 20 min was 52.9%, and after 60 min a complete loss of activity was observed.

The results indicate that the selected organism *Penicillium* sp. SS1 is capable of growing under high alkaline conditions, and is able to use agriculture-based crude carbon and nitrogen sources for the production of xylanase. The xylanase so produced is highly alkalistable and thermostable. Considering vast biotechnological applications of alkalistable and thermostable xylanases, this xylanase may be of potential commercial significance. Further studies on the organism and its xylanase must be carried to better understand the organism and the enzyme.
